# Molecular Biology of Ovarian Cancer: From Mechanisms of Intraperitoneal Metastasis to Therapeutic Opportunities

**DOI:** 10.3390/cancers13071661

**Published:** 2021-04-01

**Authors:** Krzysztof Książek

**Affiliations:** Department of Pathophysiology of Ageing and Civilization Diseases, Poznań University of Medical Sciences, Długa 1/2 Str., 61-848 Poznań, Poland; kksiazek@ump.edu.pl

Ovarian cancer (OC) is one of the most frequent malignancies of the female genital tract, and is still the leading cause of death from gynecological tumors [1]. Although the pathogenesis of ovarian cancer is well recognized, including the nature of initiating agents and mechanisms of primary and metastatic tumor development, some issues still hamper a full understanding of the complexity of this disease. These gaps may, at least partly, explain the outcomes of OC therapy, which are far from satisfactory. As a matter of fact, despite well-established algorithms of treatment [2,3], approximately 70% of ovarian cancer patients relapse within 2 years after primary cytoreduction and first-line chemotherapy [4]. Having all the above considerations in mind, prominent experts in OC pathobiology were invited to contribute to the Special Issue of Cancers devoted to molecular aspects of OC progression and therapy. The series consists of 11 excellent articles, including 9 original studies and 2 reviews.

One of the most significant findings regarding high-grade serous ovarian cancers (HGSOC), being one of the most frequent and aggressive OC histotypes [5], is that they originate from the fallopian tube [6]. The transformation of the fallopian tube epithelium (FTE), providing HGSOCs precursors, has previously been linked with the IGF-1R/AKT pathway, activated by constituents of ovulatory follicular fluid [7]. Simultaneously, molecular mechanisms of their transfer towards the ovaries and peritoneum are still a matter of investigation. A significant contribution to this research comes from the observations of Hsu and colleagues, who showed that the follicular fluid plays a causative role in the whole sequence of events leading from HGSOC formation to their intraperitoneal metastasis. Namely, the fluid supports a broad spectrum of aggressive behaviors of transformed FTE and HGSOCs, such as: migration, anchorage-independent growth, proliferation, invasion, peritoneal adhesion, and anoikis insensitivity [8]. 

HGSOCs were also among tissue and cellular OC models in the study of Batool et al., focused on the biological role of the CD83 molecule. The role of this glycoprotein, which exists in both membrane-bound and soluble forms and controls the reactivity of numerous immune cells [9], has never been tested with respect to OC properties and development. These authors addressed this challenge and demonstrated that CD83 hyperexpression promotes proliferation, colony formation, spheroid formation, and in vivo tumorigenicity of various representative OC cell lines, simultaneously limiting migration and invasion. The intensified cell growth and spheroidogenesis have been found to be mediated by CD83-dependent activation of MAP3K7-MEK1/2-ERK1/2 cascade, leading to the modulation of their downstream FOXO1/p21/CDK2/CCNB1 and STAT3/DKK1 signaling routes [10]. These studies portray the CD83 molecule as an essential regulator of OC progression, and a potential target for therapeutic interventions. The same conclusions may apply to the findings provided by Pakuła and co-workers, who described, as the first worldwide, that primary OC cells obtained from patients who had not received chemotherapy may undergo spontaneous, replicative senescence [11]. In this paper, the authors comprehensively describe the molecular mechanisms of OC cell senescence, and demonstrate that this process may be, at least partly, induced by normal peritoneal cells (mesothelium and fibroblasts), as well as by constituents of malignant ascites. Taking into account that cellular senescence is an example of a two-edged sword (limiting proliferation of a senescent cell but promoting, at the same time, the growth of its cancerous bystanders) [12], the biological and clinical significance of OC cell senescence remains to be explored. 

Immunologic status of a tumor microenvironment is critical for both OC progression and its therapy [13]. According to the current knowledge, immune cells, e.g., tumor-infiltrating CD8+ T cells, modulate the survival of OC patients [14]. Westergaard et al. addressed the problem of the immune contexture in matched primary and recurrent OCs, and found that the disease progression is associated with the development of adaptive immune resistance. At the same time, they discovered that recurrent tumors display increased immune and stromal cell infiltration (e.g., cytotoxic CD8+ T cells, helper CD4+ T cells, tumor-associated macrophages) along with the expression of immune suppressive markers [15]. These findings indicate that more inflamed recurrent ovarian tumors seem to be vulnerable to immunotherapy, and genes shown to be upregulated, *LAG3*, *HAVCR2*, *TIGIT*, and *CTLA4,* could be taken into account as promising targets. 

The importance of immune cells, specifically neutrophils, in the OC microenvironment was also tested by Muqaku and colleagues [16]. The foundation for this study was the conflicting information regarding the role (harmful vs. desirable) of these cells in OC progression [17,18]. The study revealed that neutrophils activity, marked by the formation of neutrophil extracellular traps (NETs) [19], is associated with the non-miliary spread of HGSOC, which is characterized by better overall survival, despite more invasive behavior (compared with the miliary type). Taking into account this clear contradiction, the authors of this report propose a scenario explaining the beneficial role of NETs in OC patients, which includes its initiation by peritoneal hypoxia, the establishment of distinct mascroscopic features related to a specific NET-related biomarker profile, and the modulation of the adaptive immune system by NET, which leads to improved overall survival [16].

Another element of the tumor microenvironment, apart from immune cells, are cancer stem cells. Importantly, they are usually therapy-resistant and responsible, at least to some extent, for OC relapse. In their study, Terraneo et al. show that the L1 cell adhesion molecule (L1CAM) is a new OC stem cell marker, responsible for tumor insensitivity to radiation and the high tumor-initiating capacity of the stem cells. These observations direct our attention to a specific population of OC-associated cells, namely L1CAM+/CD133+ cells, which may determine some tumor-promoting phenotypes and represent a target for novel therapeutic approaches in OC patients [20].

Some summary of the threads related to the importance of the tumor microenvironment in OC development is the review paper by Hassan et al., in which the role of selectins as mediators of OC cell adhesion to normal peritoneal cells is widely presented and discussed [21].

As per potential therapeutic solutions, van den Brand and colleagues show that epithelial cell adhesion molecule (EpCAM)-directed designed ankyrin repeat proteins (DARPins) combined with the photosensitizer IRDye 700DX may be attractive targets for photodynamic therapy in OC patients. Notably, employment of this modality resulted in a significant reduction of vitality within OC cell lines (OVCAR-3, OV90, SKOV-3), 3D tumor spheroids, and mice xenografts. The conjugates also expressed specific binding in primary patient samples [22]. Brückner et al. revealed, in turn, that the exposure of OC specimens to a transcription factor FOXM1 inhibitor (thiostrepton) and carboplatin or olaparib improves treatment response compared to single agent effects. The authors of this study also present their point of view that investigations of tumor survival using tissue cultures represent a more differentiated and comprehensive model than experiments based on conventional cell cultures [23]. A very intriguing therapeutic approach is also described in the study by Stump and collaborators, who provide a concept that resected and inactivated OC tissue could be used as a source of personalized vaccine antigen to create systemic antitumor immunity, preventing relapse. The study is focused on a mouse model to optimize the whole procedure. Cowpea mosaic virus (CPMV) was used as an adjuvant in a prophylactic vaccine, and its co-delivery with irradiated ovarian cancer cells appeared to be an effective preventive vaccine against murine OC [24]. To conclude considerations regarding modern therapeutic options for OC patients, Yang and colleagues provide a review of the literature on the importance of obesity in the development and progression of the disease. In this context, the authors also refer to recent studies describing the importance of sterol regulatory element binding protein 1 (SREBP1), a transcription factor controlling de novo lipogenesis and lipid homeostasis, and responsible for increased lipogenesis in cancer cells, as well as their elevated proliferation and metastasis [25]. 

Taken together, in this Special Issue, we were able to gather an exciting mix of articles dealing with either already opened or completely new avenues in OC research. As co-editor of this Special Issue, I am convinced that the publications contained herein will be of great interest to basic scientists and clinicians, translating into further development of the research threads described above. Personally, I note with great satisfaction the apparent increase in the proportion of research conducted using primary material from OC patients instead of established, permanent cell lines. This desirable trend should translate into a greater value of findings, from those obtained in the laboratory under in vitro conditions to the complex and individual-specific situation observed in human organisms in vivo. 
